# An Evaluation of Systematic Tuberculosis Screening at Private Facilities in Karachi, Pakistan

**DOI:** 10.1371/journal.pone.0093858

**Published:** 2014-04-04

**Authors:** Jacob Creswell, Saira Khowaja, Andrew Codlin, Rabia Hashmi, Erum Rasheed, Mubashir Khan, Irfan Durab, Christina Mergenthaler, Owais Hussain, Faisal Khan, Aamir J. Khan

**Affiliations:** 1 Stop TB Partnership, Geneva, Switzerland; 2 Interactive Research and Development, Karachi, Pakistan; Health Canada and University of Ottawa, Canada

## Abstract

**Background:**

In Pakistan, like many Asian countries, a large proportion of healthcare is provided through the private sector. We evaluated a systematic screening strategy to identify people with tuberculosis in private facilities in Karachi and assessed the approaches' ability to diagnose patients earlier in their disease progression.

**Methods and Findings:**

Lay workers at 89 private clinics and a large hospital outpatient department screened all attendees for tuberculosis using a mobile phone-based questionnaire during one year. The number needed to screen to detect a case of tuberculosis was calculated. To evaluate early diagnosis, we tested for differences in cough duration and smear grading by screening facility. 529,447 people were screened, 1,010 smear-positive tuberculosis cases were detected and 942 (93.3%) started treatment, representing 58.7% of all smear-positive cases notified in the intervention area. The number needed to screen to detect a smear-positive case was 124 (prevalence 806/100,000) at the hospital and 763 (prevalence 131/100,000) at the clinics; however, ten times the number of individuals were screened in clinics. People with smear-positive TB detected at the hospital were less likely to report cough lasting 2–3 weeks (RR 0.66 95%CI [0.49–0.90]) and more likely to report cough duration >3 weeks (RR 1.10 95%CI [1.03–1.18]). Smear-positive cases at the clinics were less likely to have a +3 grade (RR 0.76 95%CI [0.63–0.92]) and more likely to have +1 smear grade (RR 1.24 95%CI [1.02–1.51]).

**Conclusions:**

Tuberculosis screening at private facilities is acceptable and can yield large numbers of previously undiagnosed cases. Screening at general practitioner clinics may find cases earlier than at hospitals although more people must be screened to identify a case of tuberculosis. Limitations include lack of culture testing, therefore underestimating true TB prevalence. Using more sensitive and specific screening and diagnostic tests such as chest x-ray and Xpert MTB/RIF may improve results.

## Introduction

While considerable progress in detection and notification of tuberculosis (TB) was seen in the 1990s and the early part of the last decade, the number of people diagnosed and notified with TB has stalled, and three million people every year are not identified and treated by National TB Programmes (NTP) [1]. Of the millions of people who are estimated to go without a proper diagnosis and treatment each year globally, many do not have good access to services [2], are not identified when they do access care [3], or remain undetected even after being tested for TB due to insensitive diagnostics [4] and/or improper methods [5]. In many Asian settings, a large proportion of people with TB are detected and treated by private providers, and unless their clients are linked to quality diagnosis and treatment, case notification is absent and suboptimal outcomes may occur [6]. In 2006 the World Health Organization (WHO) adopted the Stop TB Strategy which recognized the importance of engaging all care providers [7]; some impressive achievements in case detection have been made using this approach [1], [8]. In Pakistan, even though the NTP reports very high treatment success among cohorts of TB patients, many people with TB symptoms and disease still attend private clinics for diagnosis and treatment and are thus missed by the singular focus on public facilities and centers operated by non-governmental organizations [9]. For the patient visiting private facilities, this can mean higher costs of treatment and sub-optimal care, outside of NTP purview [10].

While there are clearly many people with TB who access private care, little is known about what screening approaches may yield more cases in the private sector, or how to find them earlier in the course of their disease. Historically, approaches to engaging the private sector have relied on training and sensitizing private providers and asking referral from patients identified in their routine practice, similar to passive case finding approaches in the public sector [11], [12]. Increasingly however, attention is being given to active case finding approaches to improve both case notifications and the earlier detection of those cases [13]–[15].

The number of people needed to screen (NNS) provides a basic indicator of the efficiency of an intervention and a measure of potential to improve case notification in a given population. A systematic review of the NNS to detect an active case of TB found large variation across a few published papers on screening in general outpatient settings from high burden countries, and found no data on screening in private facilities [16]. While indiscriminate mass screening in low burden settings has been documented and determined to be ineffective [17], [18], there have been only two studies comparing systematic screening in hospitals and primary care facilities, both of which were carried out in public facilities in India over short periods of time [19], [20]. To measure early case detection, evaluating smear grading is a useful approach when using microscopy as the diagnostic test [21]. However there have been no publications documenting the differences in early case detection between primary, secondary or tertiary care facilities, nor any looking at both early and increased detection in the private sector.

The objectives of this study were to assess the effectiveness of systematic screening for TB in different types of private facilities in Karachi, Pakistan; to describe the characteristics of people with symptoms of TB and confirmed cases of TB and how they differ by the place of care seeking; and to evaluate the opportunities for early case detection across the facilities.

## Methods

We retrospectively assessed the yields of screening for TB symptoms and risk factors at private healthcare facilities in Karachi, Pakistan. The intervention was implemented in two towns over a one year period (January 1 to December 31, 2012). Screening of all outpatients was implemented at 44 general practitioner (GP) clinics and the outpatient department (OPD) of one large reference hospital in Korangi town, and in another 45 GP clinics in the adjacent town of Landhi. The clinics were selected to increase the numbers of clients reached rather than obtain a random sample of all GP clinics in the area. We tried to select high volume clinics (more than 50 clients per day). Not all GP clinics were open for the entire study period, as some sites closed and others opened during the study duration. The total estimated population in the two towns was 2.13 million in 2011.

All GPs were registered medical practitioners who generally worked alone in small private clinics with a single room waiting area. GPs generally charged between 5–150 Pakistani Rupees (0.05–1.77 USD) for a consultation fee. Screeners, trained community lay workers, were paid a basic monthly stipend and those at GP clinics received a series of cash incentives for: completing a daily screening report, collecting good quality sputum samples, identifying a sputum smear positive (SS+) case, and identifying a smear negative pulmonary TB case. Screeners at the OPD were paid a fixed monthly salary. Most clinics were open no less than 12 hours a day; screeners worked at least one eight hour shift per day but some worked longer.

### Screening methods

The screening procedures for all clinics and the OPD were identical. Screeners worked in the waiting area of the sites and approached all patients and their attendants. They administered a quick screen through a mobile phone application to identify people for further diagnostic testing. Anyone with a cough of 3 weeks or more in duration (or 2–3 weeks of productive cough), a previous history of tuberculosis, or a family member currently diagnosed with the disease was suspected to have tuberculosis. Verbal consent was requested; further personal details were obtained and then the individuals were asked to submit two sputum samples for smear microscopy. A spot sample was requested from all suspects after the screening questionnaire was completed. In addition, a second sputum container was provided so suspects would return with a morning specimen the following day as per NTP guidelines. Screeners received a small incentive for each sputum sample submitted, ensuring they would follow up on individuals who had yet to submit two samples. Project monitors visited the GP clinics each night to transport sputum samples to the laboratory where they were examined the following morning. In addition, the attending physician could designate an individual as someone with suspected extra pulmonary TB which would also be captured on the mobile phone form. Among people identified for further testing, the screener then captured information on other TB compatible symptoms including haemoptysis, fever, weight loss and night sweats.

### Diagnostic procedures

All people identified for smear microscopy, including those who could not produce sputum, were requested to present to the hospital OPD or mobile unit for a free chest x-ray (CXR). All CXRs were read by trained radiologists and graded as suggestive of TB, suspicious of TB, other abnormality or normal.

Two specimens collected at GP clinics and the hospital OPD (spot and morning) were transported daily by study personnel for testing according to NTP guidelines at the Indus Hospital laboratory. The laboratory is accredited by the Pakistan National Reference Laboratory through routine external quality assurance reporting. Sputum quality was assessed by the laboratory personnel and specimens which consisted of mostly saliva or were of insufficient volume were not accepted. As per the NTP guidelines for TB diagnosis, any person with at least one positive smear result (inclusive of scanty readings) was considered SS+ and eligible for treatment. If multiple slides had different results, the highest smear grading was used for classification. Treatment was provided free of charge with NTP provided drugs. Although children were screened, we only included those 10 years or older in our analysis, as many younger children have a difficult time producing quality sputum samples.

### Data collection and analysis

All data were collected using a custom-built software loaded onto GPRS enabled, Java platform mobile phones and were securely transmitted to a mySQL database over a mobile network. De-identified data were abstracted from the project database and analyzed using Stata/IC version 11. The number needed to screen (NNS) to find a smear-positive and all forms TB case and the yield of microscopy were calculated for each quarter and stratified by different intervention sites. We also tested for differences in smear grading by gender and screening facility. We tested associations for significance using Pearson chi-square two-tailed test, and calculated risk ratios (RR). This study was reviewed and approved by the institutional review board at Interactive Research and Development. Informed consent was taken verbally from all individuals who had suspected TB and was documented when the screener signed and dated the consent forms. Verbal consent was administered to adults and to parent/legal guardians of children. Written consent was not obtained because a large proportion of the target population is illiterate or semiliterate and requiring individuals to sign a document they are unable to fully understand has the potential to raise unnecessary fear and suspicion. The IRD institutional review board approved this consent procedure for adults and children.

## Results

During the study period, 541,366 people attended the screening sites and 529,447 (97.8%) individuals were verbally screened across all intervention sites (Table 1). Screening at GP clinics accounted for 482,498 (91.1%) of all individuals screened while screening at the hospital OPD accounted for the remaining 46,949 (8.9%). Verbal screening yielded 16,908 (3.2%) individuals with suspected TB, of whom 13,366 (79%) were identified at GP clinics and 3,542 (21%) at the hospital OPD. TB was suspected (screened positive) in 7.5% of people screened at the hospital OPD and 2.8% at the GP clinics. Of those who screened positive, 11,069 (65.5%) individuals submitted sputum for testing and 1,010 (9.1%) of them had SS+ TB. In addition to the 1,010 SS+ patients, 857 people were diagnosed with other forms of TB after chest X-ray and clinical evaluation, including 223 individuals with extrapulmonary TB, and 634 with sputum smear negative TB. Sample collection rates differed between screening sites (81.7% in the hospital OPD vs. 61.2% in the clinics. The overall NNS to find a person with SS+ TB was 524. However, when disaggregated by intervention sites, the NNS was 763 at GP clinics and 124 at the hospital OPD (Table 1). The NNS to find a patient with all forms TB was 284, with large differences between GP clinics and the hospital OPD (383 vs 77). There were large quarterly variations in both facility types in the NNS for SS+ TB (range 634–1,005 for clinics and 88–176 for the hospital OPD) as well as all forms TB (range 287–582 for clinics and 58–106 for the hospital OPD). The lowest NNS recorded at the hospital OPD was in the fourth quarter, while the lowest SS+ NNS at GP clinics was reported in the second quarter. Of the 1,010 SS+ cases identified, 942 (93.3%) initiated treatment; while from the number of all forms of TB cases, 1,765 (94.5%) of the patients diagnosed were started on treatment. Thirty four (33.3%) of the 102 patients with all forms of TB who did not start treatment were identified at the hospital OPD.

**Table 1:** Results of tuberculosis screening of private health facility attendees in Karachi, 2012.

Hospital Outpatient Department	Q 1		Q 2		Q 3		Q 4		Total	
	N	(%)	N	(%)	N	(%)	N	(%)	N	(%)
Total Screened	13,684		14,747		10,891		7,627		46,949	(8.9%)
People with positive screen (%)	1,163	8.5%	862	5.8%	831	7.6%	686	9.0%	3,542	7.5%
People tested with SSM (%)	963	82.8%	711	82.5%	711	85.6%	508	74.1%	2,893	81.7%
Total Smear Positive	104		84		103		87		378	
Yield	10.8%		11.8%		14.5%		17.1%		13.1%	
NNS SS+	132		176		106		88		124	
Total All Forms TB	180		139		157		131		607	
NNS All Forms	76		106		69		58		77	

General Practitioner Clinics	Q 1		Q 2		Q 3		Q 4		Total	
	N	(%)	N	(%)	N	(%)	N	(%)	N	(%)
Total Screened	109,930		126,835		128,203		117,530		482,498	(91.1%)
People with positive screen	2,867	2.6%	4,047	3.2%	3,257	2.5%	3,195	2.7%	13,366	2.8%
People tested with SSM	1,994	69.6%	2,685	66.3%	2,235	68.6%	1,262	39.5%	8,176	61.2%
Total Smear Positive	134		200		181		117		632	
Yield	6.7%		7.4%		8.1%		9.3%		7.7%	
NNS SS+	820		634		708		1,005		763	
Total All Forms TB	383		416		259		202		1,260	
NNS All Forms	287		305		495		582		383	

TB  =  Tuberculosis.

SSM  =  Sputum smear microscopy.

NNS  =  Number needed to screen.

### Patients without history of anti-tuberculosis therapy

Among people with no history of anti-tuberculosis therapy (ATT) who were tested, 53.7% (n = 5,097) were male, and there was no difference in gender between the sites (Table 2). Among those tested, the most common symptoms reported at the hospital OPD and GP clinics other than cough were fever (84.5% and 91.5% p<0.001) respectively, and weight loss (83.8% and 81.2% p = 0.006). TB suspects identified at the hospital OPD who submitted sputum were significantly more likely to have longer cough duration (>3 weeks), haemoptysis, weight loss, and contact with a family member having TB, while people identified at GP clinics were more likely to have fever or night sweats. However, none of the differences in prevalence of symptoms were significant when they were compared among SS+ cases except fever, which was more prevalent among GP attendees (96.9% vs 92.0% p = 0.001). The SS+ cases found in the hospital OPD were less likely to report cough of 2–3 weeks (14.5% vs 21.9% p = 0.008) and more likely to report cough duration >3 weeks (85.2% vs 77.3% p = 0.006).

**Table 2:** Demographic and symptom profiles of screened patients without tuberculosis treatment history, Karachi 2012.

Characteristic		Proportion among people tested with suspected TB (n = 9,487)						Smear Positive Cases (n = 854)							
	Screened Positive	Total Tested (%)	Hospital OPD n	(%)	Clinics n	(%)	P value	Hospital OPD n	(%)	Clinics n	(%)	Risk Ratio	P value	Total n (%)	
Age	**14,629**	**9,487** (64.9%)	**2,265**	**23.9%**	**7,222**	**76.1%**		**311**	**36.4%**	**543**	**63.6%**			**854**	
10–15	1,062	495 (46.6%)	141	6.2%	354	4.9%	**0.013**	19	6.1%	21	3.9%	1. 58 (0.87–2.89)	0.136	40	4.7%
16–25	4,967	3,139 (63.2%)	795	35.1%	2,344	32.5%	**0.020**	136	43.7%	244	44.9%	0.97 (0.83–1.14)	0.733	380	44.5%
26–35	3,408	2,259 (66.3%)	571	25.2%	1,688	23.4%	0.073	67	21.5%	103	19.0%	1.14 (0.86–1.50)	0.365	170	19.9%
36–45	2,132	1,456 (68.3%)	329	14.5%	1,127	15.6%	0.214	41	13.2%	70	12.9%	1.02 (0.71–1.47)	0.903	111	13.0%
>45	3,060	2,138 (69.9%)	429	18.9%	1,709	23.7%	**<0.001**	48	15.4%	105	19.3%	0.80 (0.59–1.09)	0.152	153	17.9%
Gender	**14,627**	**9,487** (64.9%)	**2,265**		**7,222**			**311**		**543**				**854**	
Male	7,650	5,097 (66.6%)	1,198	52.9%	3,899	54.0%	0.361	160	51.4%	259	47.7%	1.08 (0.94–1.24)	0.292	419	49.1%
Female	6,977	4,390 (62.9%)	1,067	47.1%	3,323	46.0%		151	48.6%	284	52.3%	0.93 (0.81–1.07)	0.292	435	50.9%
Cough Duration	**14,239**	**9,432** (66.2%)	**2,239**	98.9%	**7,193**	99.6%		**310**	99.7%	**543**	100.0%	0.997 (0.99–1.00)	0.364	**853**	99.9%
<*2 weeks*	216	106 (49.1%)	20	0.9%	86	1.2%	0.236	1	0.3%	4	0.7%	0.44 (0.04–3.67)	0.658	5	0.6%
*2*–*3 weeks*	4,159	2,761 (66.4%)	475	21.2%	2,286	31.8%	**<0.001**	45	14.5%	119	21.9%	0.66 (0.49–0.90)	**0.008**	164	19.2%
>*3 weeks*	9,861	6,565 (66.6%)	1,744	77.9%	4,821	67.0%	**<0.001**	264	85.2%	420	77.3%	1.10 (1.03–1.18)	**0.006**	684	80.2%
Haemoptysis	1,705	1,271 (74.5%)	392	17.3%	879	12.2%	**<0.001**	48	15.4%	86	15.8%	0.97 (0.70–1.35)	0.876	134	15.7%
Fever	12,981	8,525 (65.7%)	1,914	84.5%	6,611	91.5%	**<0.001**	286	92.0%	526	96.9%	0.95 (0.92–0.98)	**0.001**	812	95.1%
Night Sweats	9,644	6,311 (65.4%)	1,467	64.8%	4,844	67.1%	**0.043**	214	68.8%	392	72.2%	0.95 (0.87–1.04)	0.295	606	71.0%
Weight Loss	11,633	7,762 (66.7%)	1,897	83.8%	5,865	81.2%	**0.006**	283	91.0%	482	88.8%	1.03 (0.98–1.07)	0.305	765	89.6%
Contact	1,834	1,199 (65.4%)	333	14.7%	866	12.0%	**0.001**	52	16.7%	78	14.4%	1.16 (0.84–1.61)	0.357	130	15.2%

TB  =  Tuberculosis.

OPD  =  Outpatient Department.

At GP clinics, the number of self-reported TB-related symptoms per individual was positively correlated with the rates of sputum submission and smear-positivity. Only 43% of those identified with just a cough submitted sputum and the SS+ yield was 2.2%, while 72.8% of people with cough and four other TB symptoms submitted sputum and the SS+ yield was 11.7% (Table 3). At the hospital OPD, 54.3% of those screening positive reported cough and at least three other symptoms compatible with TB, while this proportion in GP clinics was 57.6%. These individuals accounted for 65.3% and 69.2% of all SS+ patients identified in the GP clinics and Hospital OPD, respectively.

**Table 3:** Yield of TB screening by symptom and site, Karachi 2012.

	Hospital Outpatient Department						General Practitioner Clinics					
No ATT history	Screened Positive	Tested with SSM (% of positive screen)	SS+	Yield (%)	% of SS+ cases	% of people tested with symptom classification	Screened Positive	Tested with SSM (% of positive screen)	SS+	Yield%	% of SS+ cases	% of people tested with symptom classification
Total Yield	2,785	2,265 (81.3%)	311	13.7%			11,844	7,222 (61.0%)	543	7.5%		
No cough	95	26 (27.4%)	1	3.8%	0.3%	1.1%	295	29 (9.8%)	0	0.0%	0.0%	0.4%
Cough only	70	59 (84.3%)	2	3.4%	0.6%	2.6%	433	186 (43.0%)	4	2.2%	0.7%	2.6%
Cough + any 1 symptom	274	227 (82.8%)	18	7.9%	5.8%	10.0%	1,230	747 (60.7%)	29	3.9%	5.3%	10.3%
Cough + any 2 symptoms	834	688 (82.5%)	87	12.6%	28.0%	30.4%	3,068	1,900 (61.9%)	134	7.1%	24.7%	26.3%
Cough + any 3 symptoms	1,282	1,056 (82.4%)	176	16.7%	56.6%	46.6%	6,104	3,840 (62.9%)	315	8.2%	58.0%	53.2%
Cough + any 4 symptoms	230	209 (90.9%)	27	12.9%	8.7%	9.2%	714	520 (72.8%)	61	11.7%	11.2%	7.2%

History of ATT												
Total Yield	757	628 (83.0%)	67	10.7%			1,522	954 (62.7%)	89	9.3%		
No cough	43	19 (44.2%)	1	5.3%	1.5%	3.0%	88	22 (25.0%)	0	0.0%	0.0%	2.3%
Cough only	15	13 (86.7%)	0	0.0%	0.0%	2.1%	42	25 (59.5%)	1	4.0%	1.1%	2.6%
Cough + any 1 symptom	74	64 (86.5%)	3	4.7%	4.5%	10.2%	98	57 (58.2%)	7	12.3%	7.9%	6.0%
Cough + any 2 symptoms	178	146 (82.0%)	16	11.0%	23.9%	23.2%	263	156 (59.3%)	12	7.7%	13.5%	16.4%
Cough + any 3 symptoms	364	306 (84.1%)	36	11.8%	53.7%	48.7%	824	539 (65.4%)	57	10.6%	64.0%	56.5%
Cough + any 4 symptoms	83	80 (96.4%)	11	13.8%	16.4%	12.7%	207	155 (74.9%)	12	7.7%	13.5%	16.2%

ATT  =  Anti-tuberculosis treatment history.

SS+  =  Sputum Smear Positive.

SSM  =  Sputum Smear Microscopy.

### Patients with history of anti-tuberculosis therapy

Among people with history of anti-tuberculosis therapy who were tested, 49.1% (777) were male, and there was no difference in gender between the sites (Table 4). Among those tested, the most common symptoms reported at the hospital OPD and GP clinics other than cough were fever (85.7% and 89.5% p = 0.021) respectively, and weight loss (84.7% and 87.4% p = 0.125). TB suspects identified at GP clinics were substantially more likely to report night sweats (78.3% vs 69.6% p<0.001), or having a TB family contact (19.5% vs 12.7% p<0.001). The only significant symptom difference that remained was fever among the identified SS+ cases, although in the reverse direction (97.0% in the hospital OPD vs 87.6% in GP clinics p = 0.042). Among SS+ cases, a significantly greater proportion of those with ATT history found at the hospital OPD were 16–25 years old (44.8% vs 29.2% p = 0.045).

**Table 4:** Demographic and symptom profiles of screened patients with TB treatment history, Karachi 2012.

Characteristic		Proportion among people tested with suspected TB (n = 2,279)						Sputum Smear Positive Retreatment Cases (n = 156)							
	Screened Positive	Total Tested (%)	Hospital OPD n	(%)	Clinics n	(%)	P value	Hospital OPD n	(%)	Clinics n	(%)	Risk Ratio	P value	Total n	(%)
Age	**2,279**	**1,582** (69.4%)	**628**	**39.7%**	**954**	**60.3%**		**67**	**42.9%**	**89**	**57.1%**			**156**	
10–15	79	37 (46.8%)	17	2.7%	20	2.1%	0.432	2	3.0%	0	0.0%	n/a	0.183	2	1.3%
16–25	631	411 (65.1%)	185	29.5%	226	23.7%	**0.011**	30	44.8%	26	29.2%	1.53 (1.01–2.33)	**0.045**	56	35.9%
26–35	544	386 (71.0%)	163	26.0%	223	23.4%	0.242	15	22.4%	21	23.6%	0.95 (0.53–1.70)	0.859	36	23.1%
36–45	377	268 (71.1%)	99	15.8%	169	17.7%	0.312	7	10.4%	17	19.1%	0.55 (0.25–1.22)	0.138	24	15.4%
>45	648	480 (74.1%)	164	26.1%	316	33.1%	**0.003**	13	19.4%	25	28.1%	0.69 (0.39–1.24)	0.211	38	24.4%
Gender	**2,279**	**1,582** (69.4%)	**628**		**954**			**67**		**89**				**156**	
Male	1,038	777 (74.9%)	322	51.3%	455	47.7%	0.163	36	53.7%	54	60.7%	0.89 (0.67–1.17)	0.385	90	57.7%
Female	1,241	805 (64.9%)	306	48.7%	499	52.3%		31	46.3%	35	39.3%	1.17 (0.81–1.70)	0.385	66	42.3%
Cough Duration	**2,148**	**1,541** (71.7%)	**609**	97.0%	**932**	97.7%	0.378	**66**	98.5%	**89**	100.0%	0.99 (0.96–1.01)	0.430	**155**	99.4%
<*2 weeks*	96	66 (68.8%)	31	5.1%	35	3.8%	0.206	2	3.0%	0	0.0%	n/a	0.180	2	1.3%
*2*–*3 weeks*	514	373 (72.6%)	154	25.3%	219	23.5%	0.423	15	22.7%	15	16.9%	1.35 (0.71–2.56)	0.360	30	19.4%
>*3 weeks*	1,538	1,102 (71.7%)	424	69.6%	678	72.7%	0.184	49	74.2%	74	83.1%	0.89 (0.76–1.05)	0.176	123	79.4%
Haemoptysis	442	352 (79.6%)	137	21.8%	215	22.5%	0.736	12	17.9%	15	16.9%	1.06 (0.53–2.12)	0.863	27	17.3%
Fever	1,999	1,392 (69.6%)	538	85.7%	854	89.5%	**0.021**	65	97.0%	78	87.6%	1.11 (1.01–1.22)	**0.042**	143	91.7%
Night Sweats	1,672	1,184 (70.8%)	437	69.6%	747	78.3%	**<0.001**	50	74.6%	75	84.3%	0.89 (0.75–1.04)	0.135	125	80.1%
Weight Loss	1,938	1,366 (70.5%)	532	84.7%	834	87.4%	0.125	62	92.5%	82	92.1%	1.00 (0.92–1.10)	1.000	144	92.3%
Contact	387	266 (68.7%)	80	12.7%	186	19.5%	**<0.001**	13	19.4%	15	16.9%	1.15 (0.59–2.26)	0.681	28	17.9%

OPD  =  Outpatient Department.

TB  =  Tuberculosis.

Among individuals screening positive at the hospital OPD, 21.3% (n = 757) had a history of ATT while at the GP clinics the proportion was 11.4% (n = 1,522) (Table 3). Sputum submission rates were 83.0% at the hospital OPD and 62.7% at the GP clinics, similar to the rates among those without history of ATT at each facility type (81.3% and 61.0% respectively). The yield of SS+ cases detected through microscopy among those tested was 10.7% in the hospital OPD and 9.3% in GP clinics. At the hospital OPD, 59.0% of those with ATT history reported cough and at least three other symptoms compatible with TB, while the proportion in GP clinics was 67.7%. These people accounted for 70.1% and 77.5% of all SS+ patients identified in the facilities, respectively.

### Smear Grading

Table 5 shows the results of the analysis of smear grading for this intervention. Among those diagnosed with SS+ TB, those identified at GP clinics were more likely to have a +1 smear grade than those identified at the hospital OPD (RR 1.24 [1.02–1.51]). A significantly smaller proportion of individuals with SS+ TB identified at the GP clinics had +3 smear results (27.7%) compared to 36.2% at the hospital OPD (RR 0.76 [0.63–0.92]). When we stratified by ATT history the results were still significant. When scanty and +1 grades were combined, GP clinics still had a significantly greater proportion of these smear grades (data not shown).

**Table 5:** Smear grade by treatment history and screening location, Karachi 2012.

	Scanty				+1				+2				+3				Total
	n	%	RR	95% CI	n	%	RR	95% CI	n	%	RR	95% CI	n	%	RR	95% CI	
**Hospital OPD**																	
*No ATT history*	44	14.1%	1		80	25.7%	1		78	25.1%	1		109	35.0%	1		311
*ATT history*	9	13.4%	1		22	32.8%	1		8	11.9%	1		28	41.8%	1		67
*Total*	53	14.0%	1		102	27.0%	1		86	22.8%	1		137	36.2%	1		378
**GP Clinic**																	
*No ATT history*	92	16.9%	1.20	(0.86–1.66)	178	32.8%	1.27	**(1.02–1.59)**	126	23.2%	0.93	(0.72–1.18)	147	27.1%	0.77	**(0.63–0.95)**	543
*ATT history*	13	14.6%	1.09	(0.49–2.40)	34	38.2%	1.16	(0.76–1.79)	14	15.7%	1.32	(0.59–2.95)	28	31.5%	0.75	(0.49–1.14)	89
*Total*	105	16.6%	1.18	(0.88–1.60)	212	33.5%	1.24	**(1.02–1.51)**	140	22.2%	0.97	(0.77–1.23)	175	27.7%	0.76	**(0.63–0.92)**	632

Bold text indicates significance at p<0.05 level.

ATT  =  Anti-tuberculosis treatment history.

OPD  =  Outpatient Department.

GP  =  General Practitioner.

RR  =  Risk Ratio.

CI  =  Confidence Interval.

## Discussion

Systematic TB screening strategies in private sector facilities using mHealth interventions implemented by incentivized lay individuals can identify large numbers of people with symptoms of TB, promote early diagnosis, and successfully link them to treatment. We needed to screen fewer people at the hospital OPD to find a case of pulmonary TB; however, we found many more TB cases and likely found them earlier in their disease progression by screening the much larger pool of people who regularly seek care in private GP clinics. Our results demonstrate the feasibility of using such a screening approach in a high burden country with the potential benefit of earlier and increased TB case detection.

Eleven other NTP reporting sites in the towns notified 1,606 SS+ cases and 4,651 all forms cases during the study period; those identified and placed on treatment through our private sector interventions represented 58.7% of all SS+ patients in the intervention towns and 37.9% of all forms TB patients. A systematic review of the proportion of cases found by active screening approaches compared to passive case finding identified only a handful of studies. Most of them were done in low burden settings, and in all but one (United States of America) the screening programme found fewer than 250 cases [21]. In contrast, we identified a high proportion of cases in a large population while diagnosing more than 1,000 previously undetected SS+ cases. Although the NNS at the hospital OPD was lower than at GP clinics, we screened ten times the number of people in GP clinics and identified twice as many patients. Symptom and risk factor screening followed by smear microscopy in the hospital OPD identified a SS+ prevalence of 0.81% of all outpatients and their attendees, though it was only 0.13% at GP clinics. However, due to the large numbers screened at GP clinics many more cases can be found by involving them in case detection initiatives.

Interestingly, during the last quarter of systematic screening the NNS to detect a SS+ patient increased dramatically at GP clinics while it dropped at the hospital OPD. We are unsure if this is seasonal, or related to sustained efforts of screening patients and exhausting the pool of prevalent cases in the population. To understand the long term impact of sustained intensive screening on TB prevalence and mortality in this type of setting, further operational research should be done as modeling suggests that significant reductions in TB mortality and incidence might be possible using this approach [22]. In published studies in Brazil [23], Kenya [24], India [19], [20] and Tanzania [25], screening at health facilities has generally been of a short duration and with much smaller numbers. The study in Tanzania looked at results over nine months, but only screened women at hospitals and had high rates of HIV, which is not the case in Pakistan. None of the aforementioned studies presented screening results over time.

Our results suggest that screening at GP clinics will reach people with TB earlier in their disease course than screening at hospital OPDs. We found longer duration of cough at the hospital OPD compared to GP clinics. Furthermore, people diagnosed with SS+ TB in GP clinics were significantly more likely to be graded +1, while those at the hospital OPD were significantly more likely to have a +3 grade. A study in Cambodia using smear grading showed that active case finding using mobile clinics found cases earlier than passively found cases [26]. In South Africa, a study found similar results when comparing active case finding to identifying cases passively at primary and tertiary care facilities, but did not analyze differences between the types of facilities [27]. Since a response concerning presence of symptoms was actively solicited from all facility attendees in the waiting areas, we are unable to compare the benefit of screening to that of a passive approach in these facilities. However, given the above results and other studies showing that a large proportion of cases are missed using passive case finding [3], we believe this intervention would have equally impressive results elsewhere.

The majority of people with suspected TB at both the hospital OPD and GP clinics presented with cough and at least three other TB compatible symptoms. This may reflect delays in patients seeking care at any facility until symptoms are quite advanced, as some studies have found [28], or it may reflect a lack of provider attention to TB symptoms until they become severe [29], [30], which systematic screening will help to address. Of note, the proportion of patients reporting cough and at least 3 other TB compatible symptoms at the hospital OPD was similar to the GP clinics although the yield of smear microscopy was quite different between them.

While screening large numbers of people can produce significant increases in cases detected it can also impact workloads for laboratories and treatment support. Programmes undertaking large scale screening activities need to keep the impact on other areas of work in mind during planning.

Limitations of the study include the fact that age was not recorded among all screened so these analyses likely overestimate the true NNS among adults and we could not adjust for age in the presentation of results. Additionally, the study did not test any people without self-reported TB symptoms nor could we confirm smear results with culture or even Xpert, so a complete analysis of yields from different screening algorithms and diagnostic was not possible and should be ideally be conducted. The selection of GP clinics was not randomized as we attempted to capture a large number of people seeking care in the private sector by focusing on high volume clinics. While not representative of GPs in Karachi, we clearly have shown that many people can be brought into the national notification system through this type of approach. We also have not conducted a proper cost effectiveness analysis for this study which is needed for early and increased case finding approaches.

Although symptom screening followed by smear microscopy is the standard of diagnostic care in Pakistan, as in almost all high burden TB countries, it is not a sensitive approach, and also carries the possibility of substantially increasing false positive cases when large numbers are tested. Modeling suggests that using a more sensitive and specific diagnostic test such as Xpert MTB/RIF will decrease the number of false positive diagnoses [13]. There is ample evidence from prevalence surveys that large gains in overall yield can be achieved by screening with chest X-rays and widening the testing to include asymptomatic people with abnormal chest X-rays, and using culture or a more sensitive diagnostic test to detect TB [31]–[33]. In Myanmar, during the 2009–2010 prevalence survey, only 41% of SS+ patients were found through symptom screening only, while CXR screening identified 99% of all SS+ cases eventually found [34]. A recent prevalence survey in Pakistan showed national level estimates of rates of smear positive TB of 219/100,000 [35] while we found 187/100,000 using a much less sensitive approach.

In urban Pakistan, systematic screening for TB at private facilities using mobile phone software and incentives for community workers is simple, acceptable to clients and providers, and can yield large numbers of previously undiagnosed TB cases. Screening at GP clinics may find cases earlier than screening in hospital OPDs although more testing will need to be done. Testing with new diagnostics and the use of more sensitive screening tests such as digital radiology may improve results. This approach may be used successfully as part of a strategy to improve early and increased case detection in Karachi and likely in other settings where an unengaged private sector provides a substantial proportion of health care.
